# Quercetin-Driven Redox Sensitization Enhances the Antitumor Activity of a Thiol-Reactive Agent through Glutathione Disruption

**DOI:** 10.1021/acsomega.6c04893

**Published:** 2026-09-21

**Authors:** Gabriela Carrasco-Torres, Erik Andrade-Jorge, Aldo Y. Tenorio Barajas, Roberto I. Cuevas-Hernández, Ramón Román-Doval, Jesus A. Aburto-Duarte, Jaime Santoyo-Salazar

**Affiliations:** † Centro de Investigación en Ciencia Aplicada y Tecnología Avanzada, CICATA Unidad Morelos, 98807 Instituto Politécnico Nacional, Boulevard de la Tecnología, 1036 Z-1, P 2/2, Atlacholoaya, Xochitepec, Morelos City 62790, Mexico; ‡ Laboratorio de Investigación en Biomedicina y Toxicología, Sección de Estudios de Posgrado e Investigación, 27740 Escuela Superior de Medicina del Instituto Politécnico Nacional, Plan de San Luis y Díaz Mirón s/n Casco de Santo Tomás, Mexico City 11340, Mexico; § Facultad de Ciencias Físicas y Matemáticas, Benemérita Universidad Autónoma de Puebla, Puebla 72000, Mexico; ∥ Tecnológico Nacional de México, Instituto Tecnológico del Valle de Etla, Abasolo S/N, Barrio del Agua Buena, Santiago Suchilquitongo, Oaxaca 68230, Mexico; ⊥ Departamento de Matemáticas, Centro de Investigación y de Estudios Avanzados del Instituto Politécnico Nacional, Ave. IPN 2508, México City 07360, Mexico; # Departamento de Física, 42576 Centro de Investigación y de Estudios Avanzados del Instituto Politécnico Nacional, Ave. IPN 2508, México City 07360, Mexico

## Abstract

Targeting redox homeostasis represents a promising strategy to overcome adaptive mechanisms that sustain cancer cell survival under therapeutic stress. Here, we evaluated a pharmacological sensitization strategy based on quercetin (Q) preconditioning followed by treatment with 3′,5′-dimaleamylbenzoic acid (DMAB), aimed at exploiting glutathione-dependent vulnerabilities in cervical cancer cells. Q and DMAB exhibited synergistic interactions across a 6 × 6 concentration matrix, with a marked leftward shift in the dose–response profile within the IC_50_-proximal range. Combined treatment was associated with significant depletion of intracellular glutathione (GSH) and a reduction in the GSH/GSSG ratio, consistent with disruption of redox buffering capacity. Functionally, Q + DMAB reduced HeLa cell viability to 23% at 48 h and markedly increased apoptotic cell death (75%), accompanied by S-phase cell cycle arrest and inhibition of cell migration. Notably, cytotoxic effects were attenuated in nonmalignant epithelial cells. *In vivo*, combined administration significantly reduced tumor burden (69%) and improved survival in a murine L5178-Y lymphoma model, without overt signs of systemic toxicity. Collectively, these results demonstrate that Q enhances the antitumor activity of DMAB through modulation of glutathione-dependent redox homeostasis and support the potential of redox-directed combination strategies in cancer therapy.

## Graphical abstract

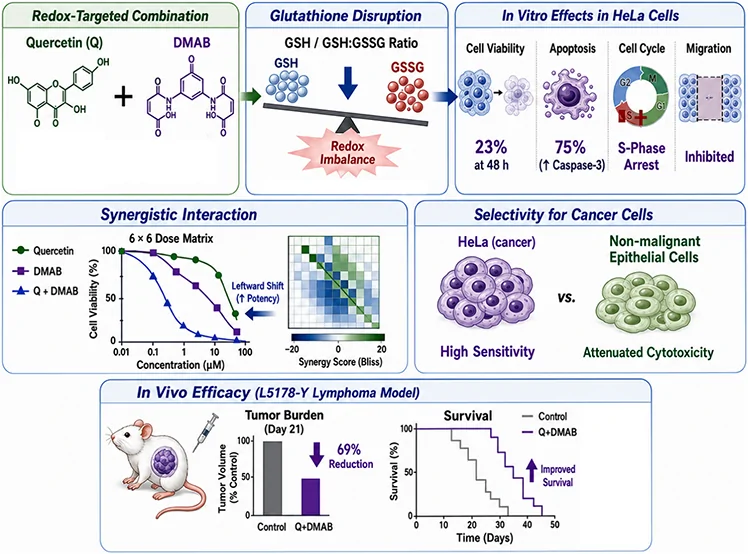

## Introduction

Cervical cancer remains a leading cause of cancer-related morbidity and mortality among women worldwide, with more than half a million new cases and approximately 350,000 deaths reported annually. This burden is particularly high in low- and middle-income countries, where limited access to screening programs and timely treatment directly impacts clinical outcomes.^1–3^ Despite advances in precision oncology and the development of targeted therapies, their application in advanced disease remains limited; therefore, it highlights the need for the development of more selective and effective methodologies and tactics.^4–6^ These limitations are largely attributed to tumor heterogeneity and the development of therapeutic resistance mechanisms,^7^ including activation of survival pathways, metabolic reprogramming, overexpression of efflux transporters, and adaptation of stress response systems, particularly those associated with the regulation of cellular redox balance.^8,9^

In this context, modulation of the intracellular redox state has been proposed as a promising strategy to increase tumor cell susceptibility to anticancer treatments. In particular, the glutathione (GSH) system plays a central role in maintaining redox homeostasis and represents one of the major cellular antioxidant buffers. Differences in the redox environment, especially those associated with GSH, can be exploited to induce selective imbalances in cancer cells and promote cytotoxic responses.^10,11^ Furthermore, the dependence of tumor cells on antioxidant systems, such as GSH-associated pathways, constitutes a critical adaptive mechanism in response to therapeutic stress and a key determinant of cell survival.^12–15^ In this regard, flavonoids have gained increasing attention in oncology due to their ability to modulate multiple processes associated with tumor biology, including proliferation, apoptosis, inflammation, metabolism, and responses to oxidative stress.^14,16–18^ Among them, quercetin (Q) stands out due to its pleiotropic profile and its capacity to modulate tumor cell behavior in a context-dependent manner. In addition to its antioxidant properties, Q has been described as a regulator of redox homeostasis and cellular sensitivity to subsequent stimuli.^19–21^ Importantly, Q has been shown to act as a pharmacological sensitizer when administered in combination with other bioactive compounds or chemotherapeutic agents, enhancing cytotoxic responses in various tumor models, including cervical cancer.^22,23^

Complementarily, thiol-reactive compounds provide a chemical strategy to modulate intracellular redox systems by engaging biological sulfhydryl groups and altering antioxidant buffering capacity. In particular, maleamide- and maleimide-derived structures can interact with −SH groups through Michael-type addition or related thiol-reactive mechanisms, enabling their interaction with glutathione, N-acetylcysteine, and cysteine-rich biomolecules, thereby affecting intracellular thiol availability and redox-dependent cellular functions.^24,25^ Previous studies with aryl maleimides, including benzoic acid-derived analogues, have shown that these compounds can reduce tumor cell viability and induce apoptosis, effects associated with glutathione depletion, increased oxidative stress, and disruption of thiol-dependent homeostasis.^26^ In addition, structurally related maleamide derivatives have demonstrated anticancer activity in different tumor models, including multitarget effects in prostate cancer and antiproliferative activity associated with caspase-3 activation in ovarian cancer cells.^27,28^ Recent studies further support the therapeutic relevance of maleimide-containing systems in oncology, showing that maleimide-functionalized nanocarriers can enhance doxorubicin delivery and antitumor efficacy, while GSH-responsive maleimide-bearing small molecules can deplete intracellular glutathione and sensitize resistant cancer cells to chemotherapy.^29–31^

In this context, 3′,5′-dimaleamylbenzoic acid (DMAB), a benzoic acid-derived compound belonging to the same maleamide- and arylmaleimide-related chemical framework previously synthesized, characterized, and evaluated for anticancer activity, represents a structurally relevant thiol-reactive agent.^24,26^ DMAB bears two maleamide-type moieties linked to a benzoic acid core, providing a chemical basis for its potential interaction with cellular thiol pools and glutathione-dependent buffering systems. Q, in turn, can be positioned not merely as a cytotoxic flavonoid, but as a redox-modulating preconditioning agent capable of reshaping the intracellular antioxidant environment before DMAB exposure and increasing tumor cell susceptibility to subsequent thiol-dependent stress.^32–34^ Based on this rationale, we hypothesized that Q-driven redox preconditioning would enhance the antitumor activity of DMAB by sensitizing cancer cells to GSH depletion and disruption of the GSH/GSSG balance. Therefore, this study evaluated a sequential Q → DMAB pharmacological strategy in cervical cancer cells and explored its antitumor efficacy *in vivo*.

## Materials and Methods

### Cell Culture and Treatments

The human cervical cancer cell line HeLa (ATCC CCL-2) was used as the tumor model. Two non-tumoral epithelial cell lines were included as controls: THLE-3 (ATCC CRL-11233), to evaluate potential off-target and systemic redox-related effects given the central role of the liver in xenobiotic metabolism, and HaCaT (ATCC PCS-200-011), as a representative epithelial model.

Cells were maintained under standard culture conditions (37 °C, 5% CO_2_, humidified atmosphere). HeLa and HaCaT cells were cultured in DMEM supplemented with 10% fetal bovine serum and 1% penicillin–streptomycin, while THLE-3 cells were cultured according to ATCC recommendations. All cell lines were thawed from cryopreserved stocks and expanded to obtain sufficient working cultures for the *in vitro* experiments. To minimize passage-related variability, HeLa and HaCaT cells were used at passage 5, whereas THLE-3 cells were used at passage 4 after thawing. Before each experiment, cultures were routinely inspected for morphology, adherence, and growth behavior, and only cells in the exponential growth phase were used. For all treatments, dimethyl sulfoxide (DMSO; Sigma-Aldrich, St. Louis, MO, USA; Cat. No. D2650) was used as vehicle control, with a final concentration not exceeding 0.1%, which did not affect cell viability.

Quercetin (Q; Santa Cruz Biotechnology, Dallas, TX, USA; Cat. No. sc-203225-A) and 3′,5′-dimaleamylbenzoic acid (DMAB) were initially evaluated in single-agent dose–response experiments. Cells were exposed to increasing concentrations (0.1–240 μM), and cell viability was assessed after 48 h to determine pharmacodynamic profiles. DMAB was synthesized and structurally characterized as previously described.^26^ Stock solutions were prepared in DMSO and diluted in culture medium immediately before use.

For drug interaction studies, a reduced concentration range (0, 0.3, 1, 3, 10, and 30 μM) was selected for both compounds. Q and DMAB were combined in a 6 × 6 factorial design (36 conditions), and cell viability was evaluated after 48 h. The resulting interaction matrix was used for synergy analysis.

For fixed-dose combination experiments, the Q → DMAB sequence was selected based on preliminary optimization assays in HeLa, HaCaT, and THLE-3 cells, in which Q pretreatment followed by DMAB produced a reproducible biological response across the evaluated cellular models. DMAB was added 30 min after Q to evaluate the potential sensitizing effect of Q on DMAB-induced cellular responses. This interval was established based on preliminary experiments assessing short-term redox modulation and was used in the subsequent mechanistic and *in vivo* studies.

### Cell Viability Assay

Cell viability was evaluated using the 3-(4,5-dimethylthiazol-2-yl)-2,5-diphenyltetrazolium bromide (MTT) assay (Thermo Fisher Scientific, Rockford, IL, USA; Cat. No. M6494) as an indicator of metabolic activity. HeLa, THLE-3, and HaCaT cells were seeded in 96-well plates at a density of 5 × 10^3^ cells per well in 100 μL of culture medium and allowed to attach overnight under standard culture conditions.

After treatment, MTT was added to each well to a final concentration of 0.5 mg/mL, followed by incubation for 4 h at 37 °C. The resulting formazan crystals were dissolved in 100 μL of dimethyl sulfoxide (DMSO), and absorbance was recorded at 590 nm using a microplate reader (iMark, Bio-Rad, Hercules, CA, USA). Background values were corrected using blank wells containing medium and MTT without cells.

Cell viability was expressed relative to vehicle-treated controls, which were set to 100%. Each condition was tested in triplicate wells, and all experiments were independently repeated three times. Data are reported as mean ± SEM, and statistical analyses were performed using GraphPad Prism (GraphPad Software, USA).

### Pharmacodynamic Modeling and Drug Interaction Analysis

Single-agent dose–response data were analyzed according to the median-effect principle described by Chou and Talalay. Cell viability values were converted into fraction affected (Fa) using

$$F_{a}=1-\frac{V}{100}$$

The median-effect equation:

$$\frac{F_{a}}{1-F_{a}}={\left(\frac{D}{D_{m}}\right)}^{m}$$

was applied to determine the slope (m) and median-effect dose (D_m_) parameters for each compound. Linear regression of:

$$\log\left(\frac{F_{a}}{1-F_{a}}\right)=m\log\left(D\right)-m\log\left(D_{m}\right)$$

was used to estimate m and D_m_.

Drug interaction within the 6 × 6 factorial matrix was evaluated using two complementary approaches.

First, Bliss independence was applied to estimate the expected additive effect:

$$E_{exp}=E_{A}+E_{B}-E_{A}E_{B}$$

Synergy was quantified as

$$\Delta E=E_{obs}-E_{exp}$$

Second, the Chou–Talalay combination index (CI) was calculated using the non-exclusive model:

$$CI=\frac{D_{1}}{(D_{x}{)}_{1}}+\frac{D_{2}}{(D_{x}{)}_{2}}+\frac{D_{1}D_{2}}{(D_{x}{)}_{1}{\left(D_{x}\right)}_{2}}$$

where D_1_ and D_2_ represent the doses used in combination to achieve a given effect level, and (D_*x*_)_1_ and (D_*x*_)_2_ represent the doses of each drug alone required to produce the same effect.

CI values were interpreted as follows: CI < 1, synergism; CI = 1, additive; CI > 1, antagonism.

All pharmacodynamic analyses were performed using custom Python scripts.

### Wound Healing Migration Assay

Cell migration was evaluated using a wound healing assay with minor modifications to the method described by Liang et al.^35^ HeLa cells were seeded in 6-well plates at a density of 3 × 10^5^ cells per well and allowed to reach 90–100% confluence.

A linear wound was created across the cell monolayer with a sterile 200 μL pipette tip. Non-adherent cells were carefully removed by washing with phosphate-buffered saline (PBS). To exclude the contribution of cell proliferation to wound closure, cells were treated with mitomycin C before exposure to the indicated treatments. Cells were then exposed to the indicated treatments, and phase-contrast images were acquired at 0 and 48 h under identical acquisition settings.

Wound closure was calculated as

$$\mathrm{Wound closure}\left(\%\right)=\frac{{\mathrm{Area}}_{0h}-{\mathrm{Area}}_{48h}}{{\mathrm{Area}}_{0h}}\times 100$$

Wound area was quantified using ImageJ software (NIH, USA). Each condition was evaluated in ten independent wells (technical replicates), and experiments were performed using independent cell preparations. Results are expressed as mean ± SEM. Statistical analyses were performed using GraphPad Prism (GraphPad Software, USA).

### TUNEL Assay for Apoptotic DNA Fragmentation

Apoptotic DNA fragmentation was assessed using a TUNEL assay (In Situ Cell Death Detection Kit, Fluorescein; Sigma-Aldrich; Cat. No. 11684795910). HeLa cells were seeded onto glass coverslips placed in 24-well plates at a density of 3 × 10^4^ cells per well and allowed to adhere overnight under standard culture conditions.

After treatment, cells were fixed with 4% paraformaldehyde for 15 min at room temperature, washed with phosphate-buffered saline (PBS), and permeabilized using 0.1% Triton X-100 in 0.1% sodium citrate for 2 min at 4 °C. Samples were then incubated with the TUNEL reaction mixture for 1 h at 37 °C in the dark. Negative controls were prepared by omitting terminal deoxynucleotidyl transferase, while positive controls were obtained by DNase I treatment.

Nuclei were counterstained with propidium iodide (PI) to visualize total cell nuclei. Coverslips were mounted using Vectashield medium and analyzed by confocal microscopy under identical acquisition settings.

TUNEL-positive nuclei were quantified as a percentage of total PI-stained nuclei using ImageJ (NIH, USA). At least five random fields per coverslip were analyzed. Each condition included technical replicates (n = 10 coverslips), and experiments were performed using independent cell preparations. Data are expressed as mean ± SEM, and statistical analysis was carried out using GraphPad Prism (GraphPad Software, USA).

### Western Blot Analysis of Caspase-3 Activation

After treatment, HeLa cells were lysed in RIPA buffer (Thermo Fisher Scientific) supplemented with protease inhibitors. Protein concentration was determined using a Bio-Rad Protein Assay (Bio-Rad, Hercules, CA, USA).

Protein extracts (30 μg per sample) were separated on 10–12% SDS–PAGE gels and transferred onto PVDF membranes. Membranes were blocked with 3% nonfat milk in PBS-T (0.1% Tween-20) and incubated overnight at 4 °C with primary antibodies against caspase-3, 1:1000 dilution (Cell Signaling Technology; Cat. No. 9662) and *β*-actin, 1:2000 dilution (Cell Signaling Technology; Cat. No. 4970). After washing steps, membranes were exposed to horseradish peroxidase (HRP)-conjugated secondary antibodies.

Signal detection was performed using enhanced chemiluminescence (ECL), and images were acquired with a ChemiDoc MP Imaging System (Bio-Rad). Band intensities were quantified using ImageJ (NIH, USA) and normalized to *β*-actin as the loading control.

All experiments were conducted using three independent biological replicates. Data are expressed as mean ± SEM, and statistical analysis was carried out using GraphPad Prism (GraphPad Software, USA).

### Cell Cycle Distribution Analysis

Cell cycle distribution was analyzed by flow cytometry following propidium iodide (PI) staining. HeLa and HaCaT cells were seeded in 6-well plates at a density of 3 × 10^5^ cells per well and allowed to adhere overnight under standard culture conditions. HeLa cells were treated with Q (10 μM), DMAB (10 μM), or Q + DMAB (3 μM + 3 μM), whereas HaCaT cells were exposed to vehicle or Q + DMAB (3 μM + 3 μM) for 48 h.

After treatment, cells were harvested, washed with phosphate-buffered saline (PBS), and incubated with RNase A (final concentration 0.2 mg/mL; Sigma-Aldrich; Cat. No. R6513) and PI (final concentration 100 μg/mL; Sigma-Aldrich; Cat. No. P4170) for 40 min at 37 °C in the dark.

DNA content was analyzed using a FACSCalibur flow cytometer (BD Biosciences, San Jose, CA, USA), and at least 10,000 events were acquired per sample. Cell cycle distribution (G_0_/G_1_, S, and G_2_/M phases) was determined using ModFit LT software (Verity Software House, USA).

Experiments were performed in four independent biological replicates. Data are presented as mean ± SEM. Statistical analyses were conducted using GraphPad Prism software (GraphPad Software, USA).

### Intracellular Reactive Oxygen Species (ROS) Measurement

Intracellular ROS levels were measured using 2′,7′-dichlorofluorescin diacetate (DCFH-DA; Sigma-Aldrich; Cat. No. D6883), a cell-permeable probe that is converted intracellularly into the fluorescent compound dichlorofluorescein (DCF).

HeLa and HaCaT cells were seeded in 96-well plates at a density of 1 × 10^4^ cells per well and cultured in Dulbecco’s modified Eagle’s medium (DMEM) supplemented with 10% fetal bovine serum (FBS) until approximately 80% confluence. Cells were washed with Hank’s balanced salt solution (HBSS) and subjected to serum-reduced conditions (DMEM containing 2% FBS) for 12 h prior to treatment.

Following 24 h exposure to the indicated treatments, cells were incubated with DCFH-DA working solution for 30 min at 37 °C in the dark. Fluorescence intensity was measured using a Fluoroskan Ascent fluorometer (Thermo Fisher Scientific) at excitation and emission wavelengths of 480 and 515 nm, respectively.

ROS levels were expressed as relative fluorescence units (RFU) and normalized to total protein content.

Experiments were performed in three independent biological replicates. Data are presented as mean ± SEM. Statistical analyses were conducted using GraphPad Prism software (GraphPad Software, USA).

### Intracellular Glutathione (GSH/GSSG) Analysis

Intracellular reduced glutathione (GSH) and oxidized glutathione (GSSG) levels were quantified in HeLa and HaCaT cells after 24 h of treatment using an o-phthaldialdehyde (OPT)-based fluorescence assay. Following treatment, cells were washed with Hank’s balanced salt solution (HBSS), harvested, and collected by centrifugation.

Cell pellets were resuspended in FEDTA buffer (0.1 M sodium phosphate monobasic, 5 mM EDTA, pH 8.0) and deproteinized by the addition of 25% phosphoric acid. Samples were centrifuged at 16,000 × g for 30 min at 4 °C, and the resulting supernatants were used for glutathione determination.

For GSH quantification, aliquots of the supernatant were reacted with OPT reagent, and fluorescence was measured at excitation and emission wavelengths of 350 and 420 nm, respectively. For selective GSSG determination, samples were pretreated with N-ethylmaleimide (NEM) to derivatize reduced glutathione prior to the OPT reaction.

Total glutathione content was calculated as the sum of GSH and GSSG concentrations. All values were normalized to total protein content. Intracellular redox status was assessed based on total glutathione levels (GSH + GSSG) and the GSH/GSSG ratio.

Experiments were performed in six independent biological replicates. Data are presented as mean ± SEM. Statistical analyses were conducted using GraphPad Prism software (GraphPad Software, USA).

### Murine L5178-Y Lymphoma Model

The murine L5178-Y ascitic lymphoma model was used to evaluate *in vivo* antitumor efficacy and survival outcomes following treatment. The model was established by intraperitoneal transplantation of viable tumor cells into male BALB/c mice.

Tumor cells were obtained from donor mice bearing established ascitic lymphoma. Male BALB/c mice (6–8 weeks of age, body weight 26–28 g) were used in all experiments.

All experimental protocols were approved by the Institutional Committee for the Care and Use of Laboratory Animals (CICUAL) (protocol no. 0254-11). Animals were provided by the Unidad de Producción y Experimentación en Animales de Laboratorio (UPEAL) at CINVESTAV, Mexico City, Mexico. Mice were maintained under controlled environmental conditions (12 h light/dark cycle, temperature of 21 °C, and 50% relative humidity), with unrestricted access to standard laboratory diet (PMI Feeds Inc., Laboratory Diet) and water.

For tumor induction, 3.2 × 10^6^ viable L5178-Y cells suspended in sterile 0.9% NaCl solution were injected intraperitoneally into recipient mice. Tumor establishment was confirmed by the development of ascitic fluid prior to treatment initiation.

### Treatment Protocol and Survival Analysis

The test compounds were administered once daily for 10 consecutive days by intragastric gavage (10 mL/kg body weight). A 0.9% NaCl solution was used as vehicle control. Dose selection was based on preliminary toxicity and efficacy studies.

Q was administered at 2 mg/kg body weight, and DMAB at 4 mg/kg body weight. These doses were selected to achieve biologically relevant and well-tolerated treatment conditions *in vivo*, while accounting for the pharmacological differences between *in vitro* exposure and systemic administration.

Cyclophosphamide (6 mg/kg body weight) was included as a positive control due to its well-established use as an alkylating agent in the treatment of lymphoid malignancies.

For sequential treatment groups, Q was administered first, followed by DMAB 30 min later within the same dosing session, to mimic the *in vitro* pharmacological sensitization strategy.

Survival was monitored daily, and survival time was recorded for each animal. Survival curves were generated using the Kaplan–Meier method and compared using the log-rank (Mantel–Cox) test. Investigators responsible for survival monitoring and end point assessment were blinded to treatment allocation. Humane end points were explicitly defined as severe abdominal distension, marked reduction in mobility or responsiveness, inability to access food or water, persistent recumbency, signs of severe pain or distress, or body-weight loss exceeding 20% relative to baseline. Only a limited proportion of animals exhibited these predefined humane-end point criteria during the experimental period. Animals meeting any of these criteria were immediately euthanized in accordance with the institutional animal-welfare procedures of UPEAL-CINVESTAV and the Mexican Official Standard NOM-062-ZOO-1999 for the production, care, and use of laboratory animals.

### Tumor Growth Evaluation

Tumor growth was evaluated based on ascitic tumor development and tumor cell burden. Ascitic fluid was aseptically collected using a sterile syringe, and tumor cell viability and concentration were determined using the trypan blue exclusion method with a Neubauer hemocytometer. Cell viability was expressed as the percentage of viable (unstained) cells relative to the total cell count.

Tumor burden was estimated from abdominal distension using an ellipsoid-based approximation, as previously described for murine ascitic lymphoma models.^36^ Tumor dimensions were obtained through external measurements of the abdominal region using a caliper. Tumor volume was calculated as

$$V=\frac{L\times W^{2}}{2}$$

where L represents the major length and W the perpendicular width.

Treatment efficacy was further evaluated by calculating tumor growth inhibition (TGI), defined as

$$\%TGI=\left(1-\frac{\Delta V_{\mathrm{treated}}}{\Delta V_{\mathrm{control}}}\right)\times 100$$

where Δ*V*_treated_ and Δ*V*_control_ represent the change in tumor volume (*V*_final_ – *V*_initial_) in treated and control groups, respectively.

Body weight was monitored throughout the experimental period as an indicator of systemic toxicity and overall health status.

### Statistical Analysis

Statistical analyses were performed using GraphPad Prism software (version 7.0; GraphPad Software, USA). Data are presented as mean ± SEM unless otherwise indicated. The number of independent biological replicates and, where applicable, technical replicates is specified in the corresponding Methods subsection and figure legend. Comparisons among multiple groups were performed using one-way analysis of variance (ANOVA) followed by Tukey’s multiple-comparison test. Survival curves were generated using the Kaplan–Meier method and compared using the log-rank (Mantel–Cox) test. For the *in vivo* experiments, 10 animals were included per group. Differences were considered statistically significant at *p* < 0.05. Statistical significance is indicated as ns, not significant; *p* < 0.05; *p* < 0.01; and *p < 0.001*.

## Results

### Pharmacodynamic Analysis Demonstrates Synergistic Interaction between Q and DMAB

To characterize the interaction between Q and DMAB, single-agent and combination dose–response relationships were first established and analyzed using nonlinear regression, followed by matrix-based interaction analyses using Bliss independence and the Chou–Talalay combination index (CI) framework (Figure 1).

**Figure 1:**
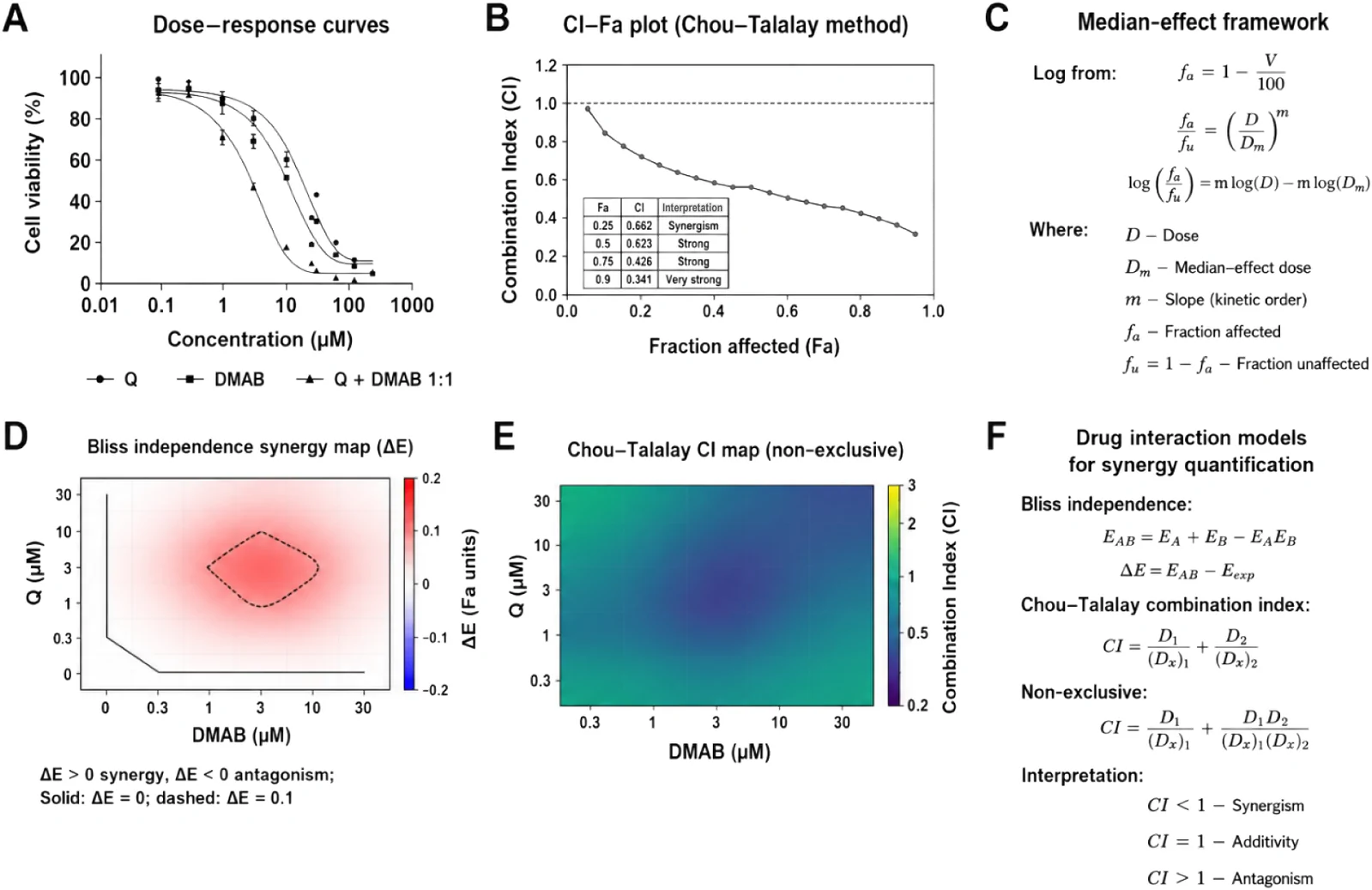
Pharmacodynamic interaction between Q and DMAB in HeLa cells. (A) Dose–response curves for Q, DMAB, and their 1:1 combination after 48 h. Viability was normalized to the vehicle-treated control and fitted using nonlinear regression (log­[inhibitor] versus normalized response). (B) Combination index–fraction affected (CI–Fa) plot based on the Chou–Talalay method. (C) Median-effect framework used for dose–response and interaction analysis. (D) Bliss independence synergy map (ΔE) across the 6 × 6 concentration matrix; positive values indicate synergy. (E) Combination index (CI) heatmap calculated using the non-exclusive model; CI < 1 indicates synergism, CI = 1 indicates additivity, and CI > 1 indicates antagonism. (F) Summary of the Bliss independence and Chou–Talalay models. Pharmacodynamic modeling was performed using mean viability values obtained from three independent biological experiments, each conducted in triplicate wells.

Q and DMAB each produced concentration-dependent reductions in HeLa cell viability, with estimated IC_50_ values of approximately 13 and 10 μM, respectively (Figure 1A). In contrast, the 1:1 Q + DMAB combination showed a marked leftward shift in the dose–response curve, with an estimated IC_50_ in the 2–3 μM range, indicating increased potency relative to either monotherapy.

To further assess drug interaction, the Chou–Talalay method was applied across increasing effect levels. The resulting CI–Fa plot showed CI values below 1 over a broad range of fractional effects, consistent with synergistic interaction (Figure 1B). Based on the single-agent response profiles, a 6 × 6 factorial concentration matrix (0, 0.3, 1, 3, 10, and 30 μM) was then designed to cover subeffective, IC_50_-proximal, and high-effect ranges.

Bliss independence analysis revealed positive ΔE values across much of the matrix, indicating that the observed combined effect exceeded the Bliss-predicted additive response (Figure 1D). This effect was most pronounced in the IC_50_-proximal region (1–3 μM). Consistently, the CI heatmap derived from the Chou–Talalay framework identified a central region of synergistic interaction, again most evident within the IC_50_-proximal range (Figure 1E).

Taken together, these results show that Q and DMAB produce a synergistic effect within a defined concentration range, with the combination exhibiting greater potency than either agent alone.

### Combined Treatment Enhances Selective, Time-Dependent Cytotoxicity in Tumor Cells

Based on pharmacodynamically relevant concentrations identified in the dose–response analysis, HeLa cervical cancer cells and the non-tumor epithelial cell lines HaCaT and THLE-3 were treated with Q (10 μM), DMAB (10 μM), or their combination (3 μM + 3 μM) for 12, 24, 36, and 48 h. Cell viability was assessed by the MTT assay.

In HeLa cells, treatment-related changes were detectable as early as 12 h. At this time point, Q and DMAB monotherapies reduced viability to approximately 87% and 90%, respectively, whereas the combination did not differ significantly from the vehicle control (Figure 2A). At 24 h, both Q and the combination produced larger decreases in viability than DMAB alone (Figure 2B). At 36 h, the combination showed the lowest viability values, decreasing the MTT-derived signal to approximately 50%, compared with about 62% for DMAB (Figure 2C). By 48 h, Q monotherapy reduced viability to approximately 54%, whereas the combination produced the strongest effect, lowering viability to approximately 23% relative to vehicle-treated cells (Figure 2D).

**Figure 2:**
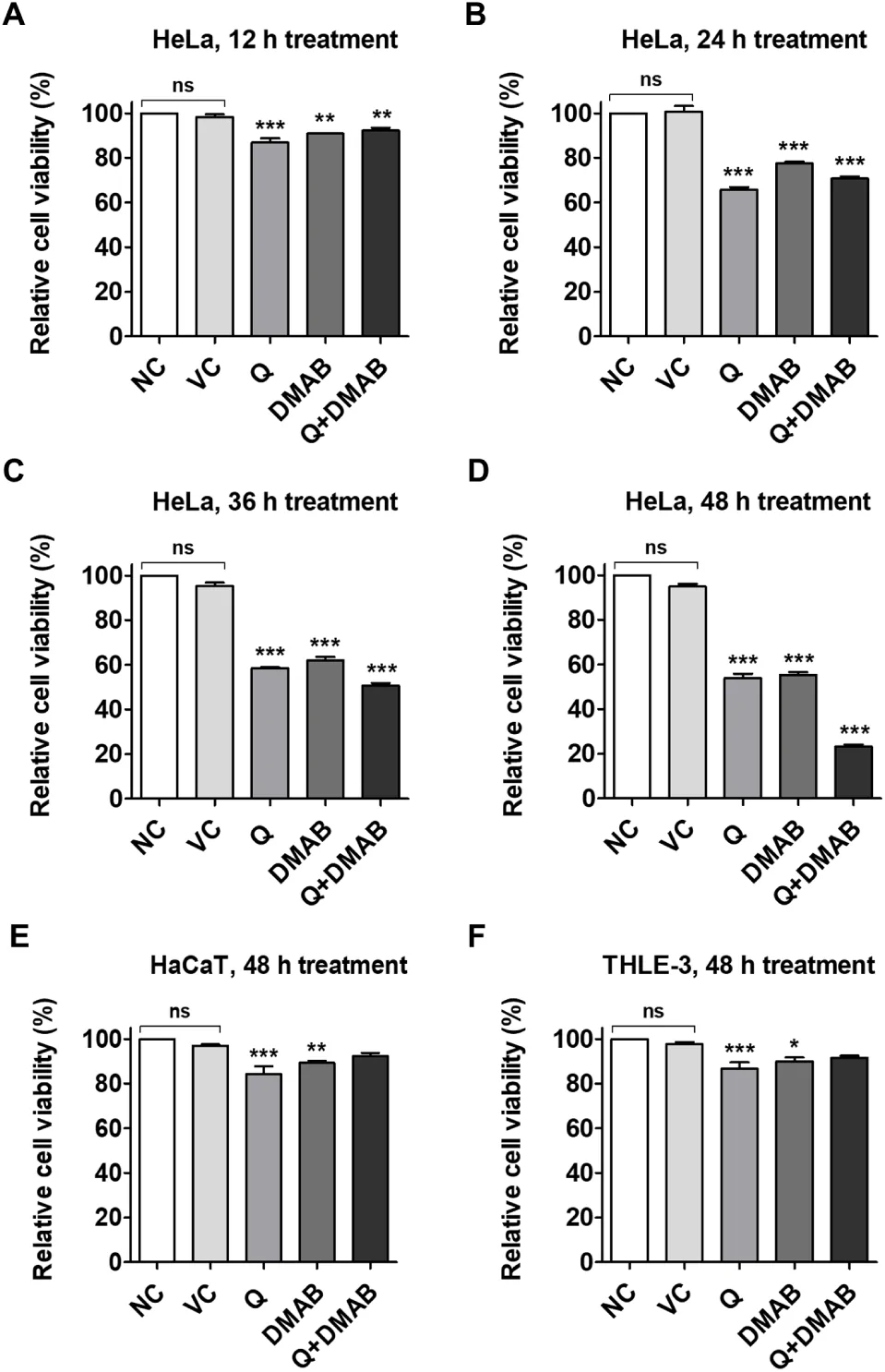
Time-dependent cytotoxic effects of Q and DMAB in tumor and non-tumor cells. HeLa, HaCaT, and THLE-3 cells were treated with Q (10 μM), DMAB (10 μM), or their combination (3 μM + 3 μM) for the indicated times. Cell viability was assessed using the MTT assay and expressed relative to the vehicle-treated control. (A–D) HeLa cells at 12, 24, 36, and 48 h, respectively. (E) HaCaT cells at 48 h. (F) THLE-3 cells at 48 h. Data are presented as mean ± SEM from three independent biological experiments, each performed in triplicate wells. Statistical significance was determined using one-way ANOVA followed by Tukey’s multiple-comparison test: ns, not significant; *p < 0.05; **p < 0.01; and ***p < 0.001 versus vehicle.

In non-tumor epithelial cells, the effects were markedly attenuated. In HaCaT cells, Q and DMAB induced modest reductions in viability at 48 h, whereas the combination produced little additional effect (Figure 2E). Similarly, THLE-3 cells showed only minor changes across treatment conditions, indicating lower sensitivity than HeLa cells (Figure 2F).

Collectively, these results indicate that Q and DMAB produce a time-dependent reduction in HeLa cell viability, with the strongest effect observed for the combination at 48 h, while non-tumor epithelial models display substantially lower sensitivity under the same conditions.

### Combined Treatment Inhibits Cell Migration and Promotes Apoptotic Cell Death

To evaluate the impact of treatment on HeLa cell behavior, wound closure and apoptosis-related end points were assessed following exposure to Q (10 μM), DMAB (10 μM), or their combination (3 μM + 3 μM) for 48 h. Wound closure was first examined using a scratch assay. Representative images are shown in Figure 3A. Control and vehicle-treated cells displayed comparable closure of the scratched area, indicating that the vehicle (0.1% DMSO) did not affect the assay. In contrast, the combination treatment resulted in a visibly wider residual wound area. Quantitative analysis showed that Q and DMAB monotherapies produced modest reductions in wound closure, whereas the combination displayed the lowest wound closure values, reaching 50% relative to control conditions (Figure 3B).

**Figure 3:**
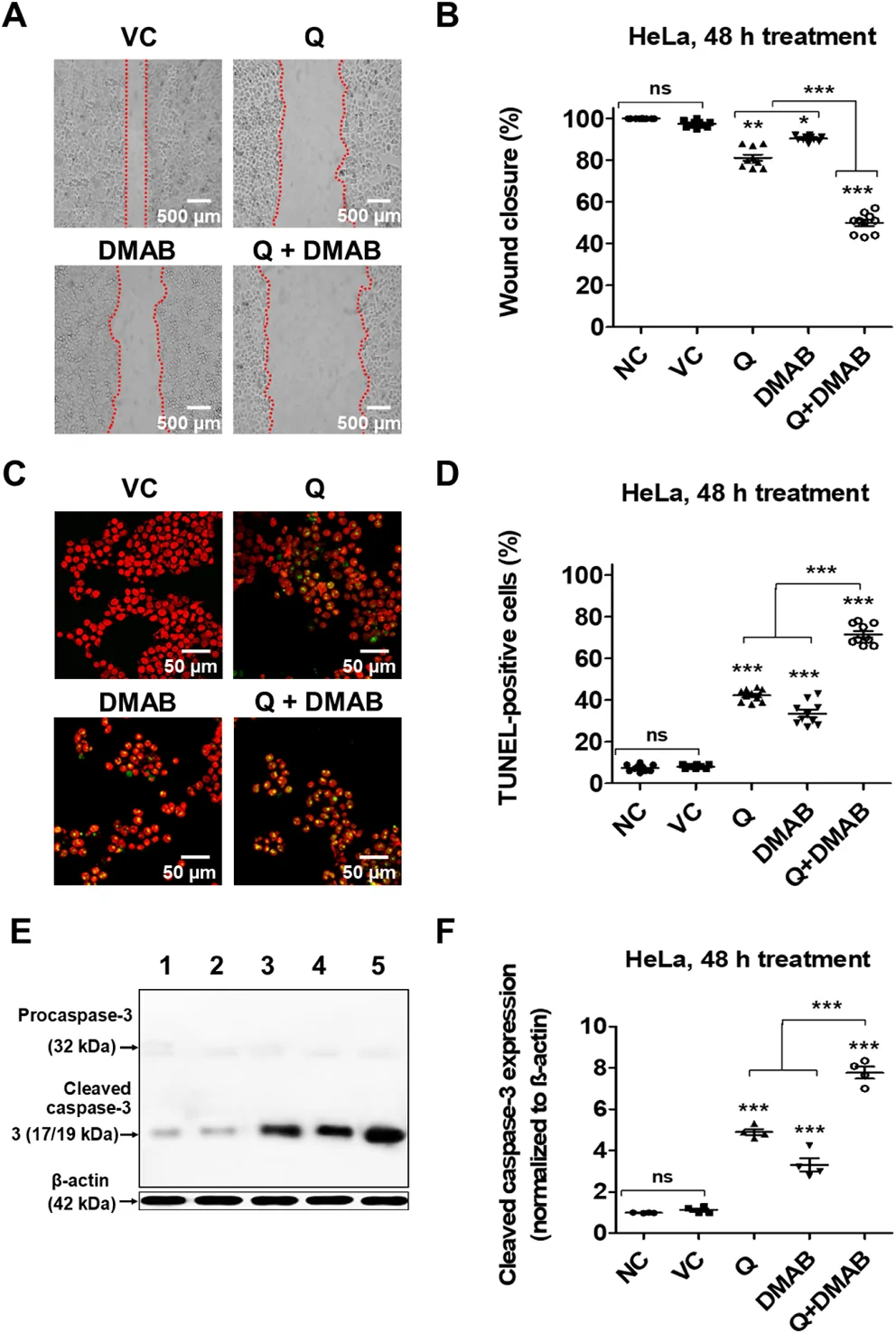
Q + DMAB reduces wound closure and promotes apoptosis in HeLa cells. (A) Representative wound-healing images of HeLa cells treated with vehicle (VC), Q (10 μM), DMAB (10 μM), or Q + DMAB (3 μM + 3 μM) for 48 h. Red dashed lines indicate the wound boundaries (4× magnification). (B) Quantification of wound closure (%). (C) Representative TUNEL images showing apoptotic nuclei (green) and total nuclei stained with PI (red); the merged yellow signal indicates TUNEL-positive nuclei (40× magnification). (D) Quantification of TUNEL-positive cells (%). (E) Representative Western blot analysis of procaspase-3, cleaved caspase-3, and β-actin. (F) Densitometric analysis of cleaved caspase-3 normalized to β-actin. Data are presented as mean ± SEM. Migration and TUNEL analyses included 10 technical replicates per condition, whereas Western blot analysis was performed using three independent biological replicates (n = 3). Statistical significance was determined using one-way ANOVA followed by Tukey’s multiple-comparison test: ns, not significant; *p < 0.05; **p < 0.01; and ***p < 0.001 versus vehicle.

To determine whether these effects were associated with apoptosis, DNA fragmentation was assessed by TUNEL staining. Representative fluorescence images are shown in Figure 3C. PI-staining identified total nuclei, whereas TUNEL-positive nuclei corresponded to apoptotic cells. Merged images indicated that TUNEL labeling was localized to PI-counterstained nuclei. Quantitative analysis revealed low basal apoptosis in control and vehicle-treated cells, while Q and DMAB increased the proportion of TUNEL-positive cells to 43% and 35%, respectively. The combination treatment produced the highest apoptotic index, reaching 70–75%, significantly above both monotherapies (Figure 3D).

To further assess the apoptotic response, caspase-3 activation was evaluated by Western blot. Both procaspase-3 (32 kDa) and cleaved caspase-3 fragments (17/19 kDa) were detected (Figure 3E). Cleaved caspase-3 levels were minimal in control and vehicle-treated cells, increased after either monotherapy, and were highest in the combination group. Densitometric analysis normalized to β-actin confirmed an approximately fivefold increase for Q, a threefold increase for DMAB, and an approximately eightfold increase for the combination relative to control (Figure 3F).

Collectively, these findings indicate that Q + DMAB markedly impairs wound closure and promotes apoptotic cell death in HeLa cells, accompanied by enhanced caspase-3 cleavage.

### Combined Treatment Induces Cell Cycle Arrest

To assess whether the antiproliferative effects of Q and DMAB were linked to changes in cell cycle progression, DNA content was evaluated by flow cytometry using propidium iodide staining. HeLa cells were exposed to Q (10 μM), DMAB (10 μM), or their combination (3 μM + 3 μM) for 48 h. Representative histograms are shown in Figure 4A, and the corresponding phase distribution is presented in Figure 4B.

**Figure 4:**
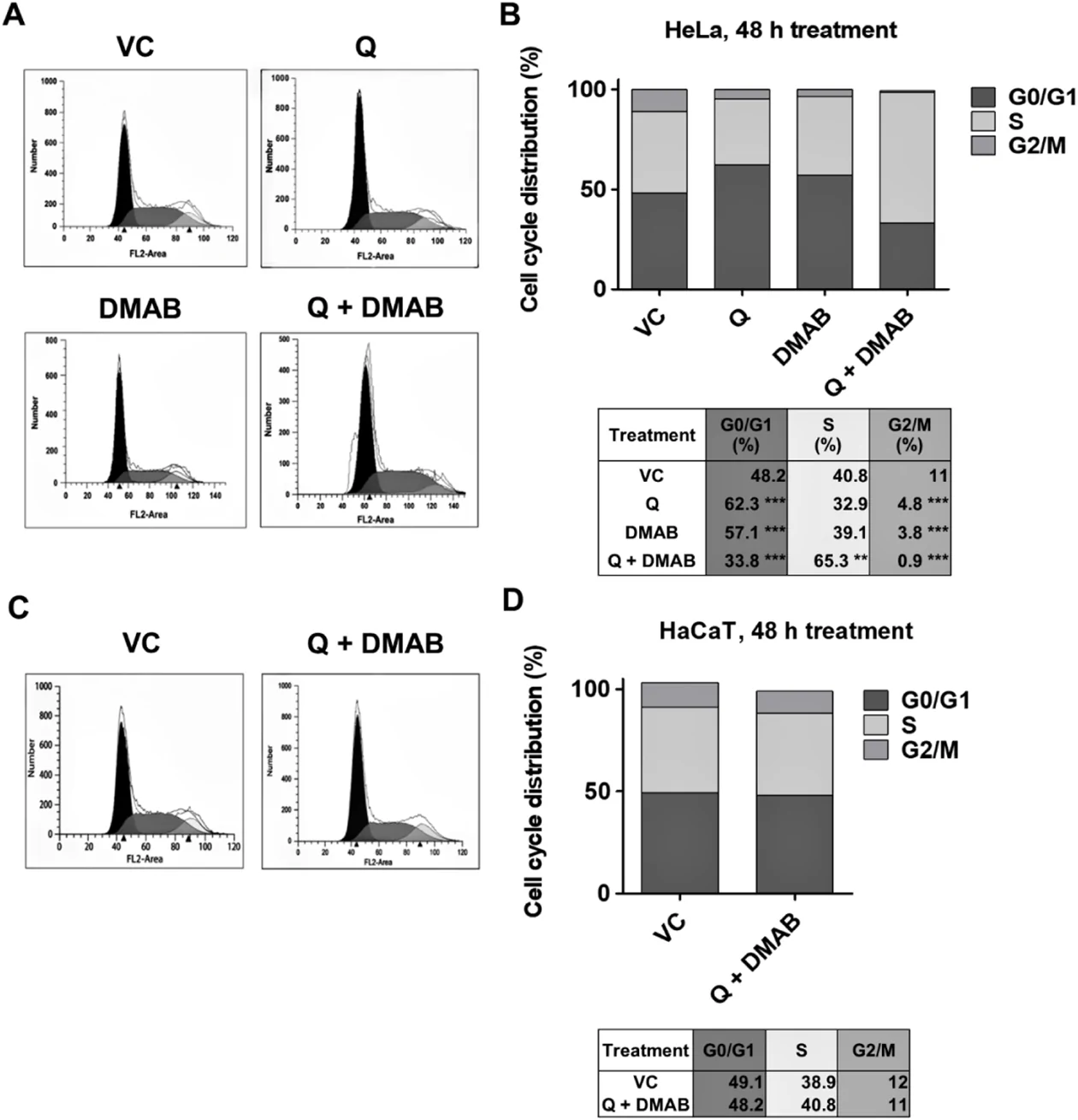
Q + DMAB differentially alters cell-cycle distribution in HeLa and HaCaT cells. (A) Representative flow-cytometry histograms of DNA content following PI staining in HeLa cells treated with vehicle (VC), Q (10 μM), DMAB (10 μM), or Q + DMAB (3 μM + 3 μM) for 48 h. (B) Quantification of the G_0_/G_1_, S, and G_2_/M phase distributions in HeLa cells; the corresponding percentages are shown in the quantitative panel. (C) Representative flow-cytometry histograms of DNA content in HaCaT cells treated with vehicle or Q + DMAB (3 μM + 3 μM) for 48 h. (D) Quantification of the G_0_/G_1_, S, and G_2_/M phase distributions in HaCaT cells; the corresponding percentages are shown in the quantitative panel. Data are presented as mean ± SEM from four independent biological experiments. Statistical significance was determined using one-way ANOVA followed by Tukey’s multiple-comparison test for HeLa cells and an unpaired two-tailed Student’s t-test for HaCaT cells: ns, not significant; *p < 0.05; **p < 0.01; ***p < 0.001 versus vehicle.

Control cells exhibited a typical cell cycle distribution, with approximately 48.2% of cells in G_0_/G_1_, 40.8% in S phase, and 11.0% in G_2_/M. Treatment with Q resulted in a marked accumulation of cells in G_0_/G_1_ (62.3%, ***), accompanied by a reduction in S phase (32.9%) and a decrease in G_2_/M (4.8%, ***), suggesting partial arrest at the G_0_/G_1_ checkpoint.

Similarly, DMAB increased the proportion of cells in G_0_/G_1_ (57.1%, ***), while reducing the G_2_/M population (3.8%, ***). The S-phase fraction (39.1%) remained comparable to control, indicating a moderate inhibition of cell cycle progression.

In contrast, the combination treatment produced a pronounced redistribution of the cell cycle profile, characterized by a substantial accumulation of cells in S phase (65.3%, **), accompanied by a marked decrease in G_0_/G_1_ (33.8%, ***) and an almost complete depletion of G_2_/M (0.9%, ***). This pattern indicates a strong S-phase arrest, consistent with impaired DNA replication or S-phase progression.

Overall, Q and DMAB as single agents mainly led to partial accumulation in the G_0_/G_1_ phase, whereas the combined treatment markedly altered cell cycle progression, promoting S-phase accumulation. This shift may be associated with the enhanced cytotoxic and pro-apoptotic effects observed.

To determine whether the S-phase accumulation induced by the combined treatment was also observed in nonmalignant epithelial cells, cell-cycle distribution was evaluated in HaCaT cells under the same treatment conditions. Vehicle-treated HaCaT cells showed 49.1% of cells in G_0_/G_1_, 38.9% in S phase, and 12.0% in G_2_/M. Following Q + DMAB treatment, the corresponding distributions were 48.2%, 40.8%, and 11%, respectively, with no significant redistribution among cell-cycle phases. These findings indicate that the marked S-phase accumulation observed in HeLa cells was not reproduced in HaCaT cells under the same experimental conditions, supporting a tumor-selective effect of the combined treatment.

### Combined Treatment Modulates Intracellular ROS Dynamics

Intracellular ROS-associated fluorescence was assessed using DCFH-DA following treatment (n = 3 independent experiments). In HeLa cells, Q significantly reduced fluorescence intensity compared with vehicle-treated controls (***), indicating a decrease in ROS-associated signal. In contrast, DMAB did not significantly alter fluorescence levels.

The combination treatment also significantly reduced fluorescence relative to control conditions (***); however, the magnitude of this reduction was lower than that observed with Q alone (Figure 5B).

**Figure 5:**
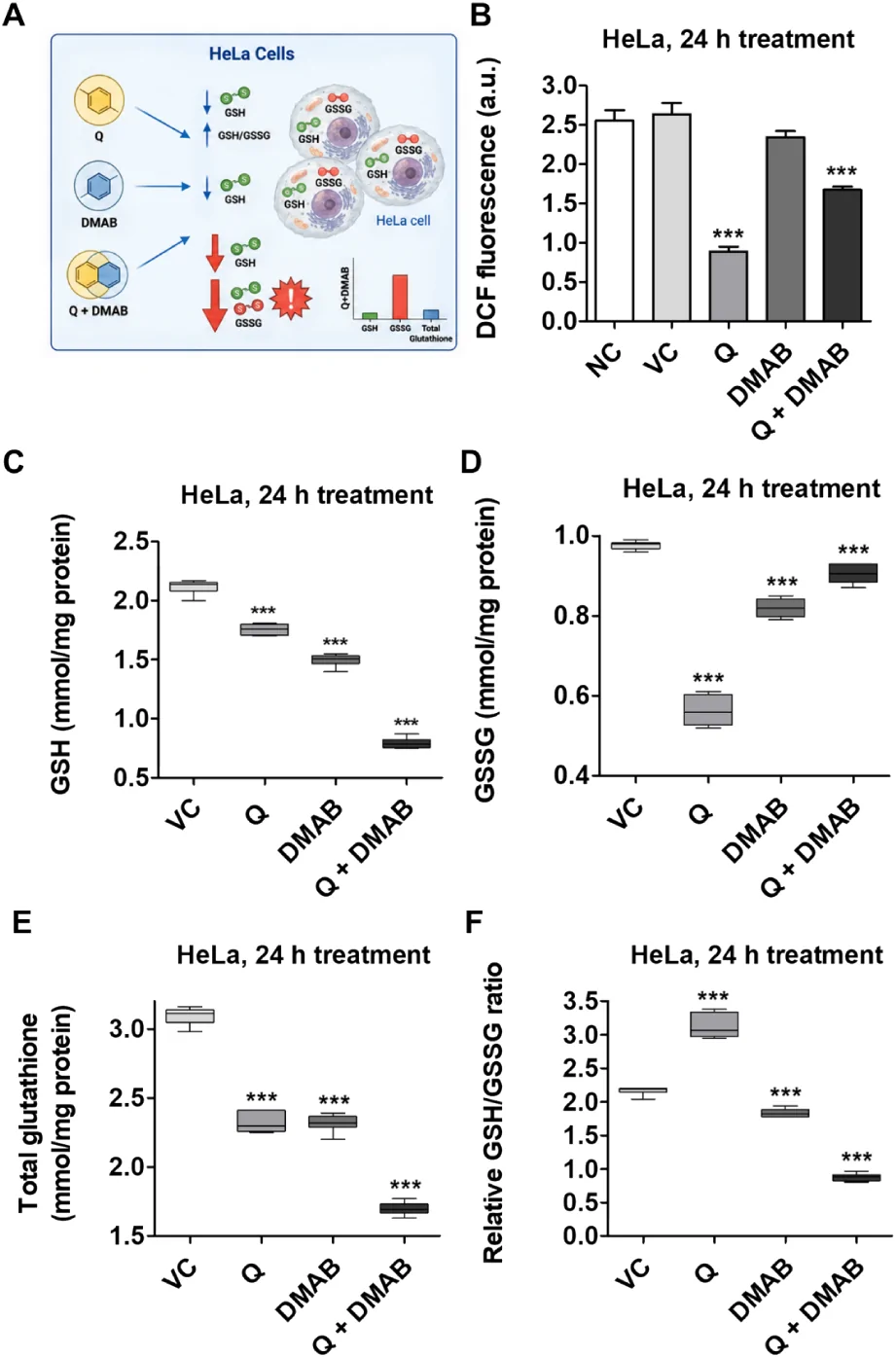
Effects of Q and DMAB on intracellular oxidative status and glutathione homeostasis in HeLa cells. (A) Schematic illustration of the redox effects induced by Q, DMAB, and Q + DMAB in HeLa cells. (B) DCF fluorescence in HeLa cells following 24 h treatment with vehicle (VC), Q (10 μM), DMAB (10 μM), or Q + DMAB (3 μM + 3 μM). (C–F) Glutathione parameters measured in HeLa cells after 24 h of treatment: (C) reduced glutathione (GSH), (D) oxidized glutathione (GSSG), (E) total glutathione, and (F) GSH/GSSG ratio. Data are presented as mean ± SEM. ROS measurements were performed in three independent biological experiments, whereas glutathione measurements were performed in six independent biological experiments. Statistical significance was determined using one-way ANOVA followed by Tukey’s multiple-comparison test: ns, not significant; *p < 0.05; **p < 0.01; ***p < 0.001 versus vehicle.

In non-tumoral HaCaT cells, Q similarly reduced fluorescence intensity compared with vehicle-treated controls (***). DMAB produced only minor changes, while the combination treatment resulted in fluorescence levels comparable to control conditions (Figure 6B).

**Figure 6:**
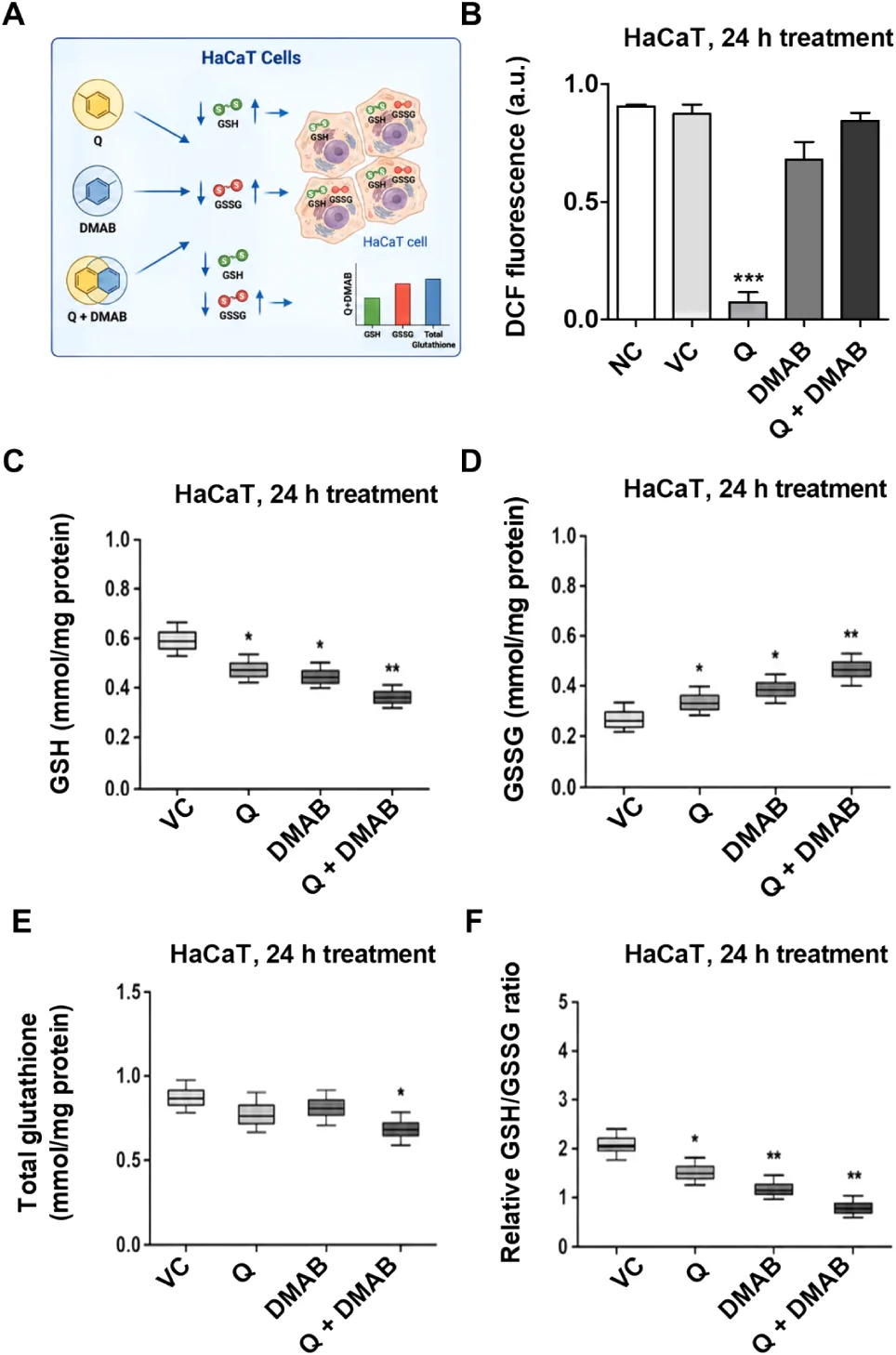
Effects of Q and DMAB on intracellular oxidative status and glutathione homeostasis in non-tumorigenic HaCaT cells. (A) Schematic illustration of the redox response induced by Q, DMAB, and Q + DMAB in HaCaT cells. (B) DCF fluorescence in HaCaT cells following 24 h treatment with vehicle (VC), Q (10 μM), DMAB (10 μM), or Q + DMAB (3 μM + 3 μM). (C–F) Glutathione parameters measured in HaCaT cells after 24 h of treatment: (C) reduced glutathione (GSH), (D) oxidized glutathione (GSSG), (E) total glutathione, and (F) GSH/GSSG ratio. Data are presented as mean ± SEM. ROS measurements were performed in three independent biological experiments, whereas glutathione measurements were performed in six independent biological experiments. Statistical significance was determined using one-way ANOVA followed by Tukey’s multiple-comparison test: ns, not significant; *p < 0.05; **p < 0.01; ***p < 0.001 versus vehicle.

### Disruption of Redox Homeostasis through Glutathione Depletion

Intracellular glutathione parameters were quantified to assess alterations in cellular redox homeostasis induced by the different treatments (n = 6 independent experiments).

In tumor cells, treatment with Q monotherapy significantly reduced intracellular glutathione pools compared with vehicle-treated controls (***), as evidenced by decreased levels of reduced glutathione (GSH) and total glutathione (GSH + GSSG). In addition, oxidized glutathione (GSSG) levels were lower than those observed in control cells, while the GSH/GSSG ratio increased, indicating a shift in intracellular redox balance in HeLa cells (Figure 5C–F).

Treatment with DMAB monotherapy also altered glutathione homeostasis in HeLa cells. GSH levels were significantly reduced (***) accompanied by a decrease in total glutathione content (***). GSSG levels were also significantly reduced (***), whereas the GSH/GSSG ratio showed a modest but significant decline (***), suggesting partial disruption of redox equilibrium in HeLa cells.

Notably, the Q + DMAB combination treatment produced the most pronounced disruption of intracellular glutathione homeostasis in HeLa cells. This treatment induced a marked depletion of reduced glutathione (GSH) (***) and total glutathione (***), together with a substantial decline in the GSH/GSSG ratio (***) relative to control conditions. GSSG levels were also slightly lower than those observed in control cells (Figure 5C–F).

To determine whether this glutathione-dependent redox disruption was also present in a non-tumorigenic epithelial model, the same parameters were evaluated in HaCaT cells after 24 h of treatment. In HaCaT cells, Q and DMAB produced moderate decreases in GSH levels, whereas Q + DMAB induced a further reduction in GSH (Figure 6C). In contrast to HeLa cells, GSSG levels increased following treatment, particularly in the Q + DMAB group (Figure 6D). Total glutathione showed only a moderate reduction compared with the marked depletion observed in HeLa cells, and the GSH/GSSG ratio also decreased, indicating redox modulation in the context of comparatively preserved total glutathione content (Figure 6E, F).

Together, these findings indicate that Q + DMAB disrupts glutathione-dependent redox homeostasis in both cellular contexts, but with a distinct pattern between tumor and non-tumorigenic cells. In HeLa cells, the combination produced marked GSH depletion, reduced total glutathione content, and a pronounced decrease in the GSH/GSSG ratio, consistent with impaired antioxidant buffering capacity. In HaCaT cells, the response was characterized by moderate GSH depletion, increased GSSG levels, and a reduced GSH/GSSG ratio, while total glutathione content was comparatively less affected. This differential response suggests that HaCaT cells retain a greater capacity to preserve the overall glutathione pool, whereas HeLa cells exhibit greater vulnerability to the combined treatment.

Overall, these results indicate that Q + DMAB primarily disrupts glutathione-dependent redox homeostasis rather than promoting detectable ROS accumulation. The pronounced reduction in GSH levels, total glutathione content, and the GSH/GSSG ratio observed in HeLa cells is consistent with compromised antioxidant buffering capacity, whereas the comparatively preserved total glutathione pool in HaCaT cells supports a differential redox response between tumor and non-tumorigenic cells.

### Combined Treatment Suppresses Tumor Growth and Prolongs Survival *In Vivo*

Tumor progression was evaluated based on ascitic fluid accumulation and viable tumor cell counts in the murine L5178-Y lymphoma model. Control groups (NC and vehicle) exhibited high ascitic fluid volumes, consistent with progressive tumor growth (Figure 7B). Treatment with DMAB and cyclophosphamide (C+) significantly reduced ascitic accumulation, whereas Q alone produced only a modest, non-significant effect. Notably, the combination treatment resulted in the greatest reduction in ascitic volume, indicating a marked suppression of tumor burden.

**Figure 7:**
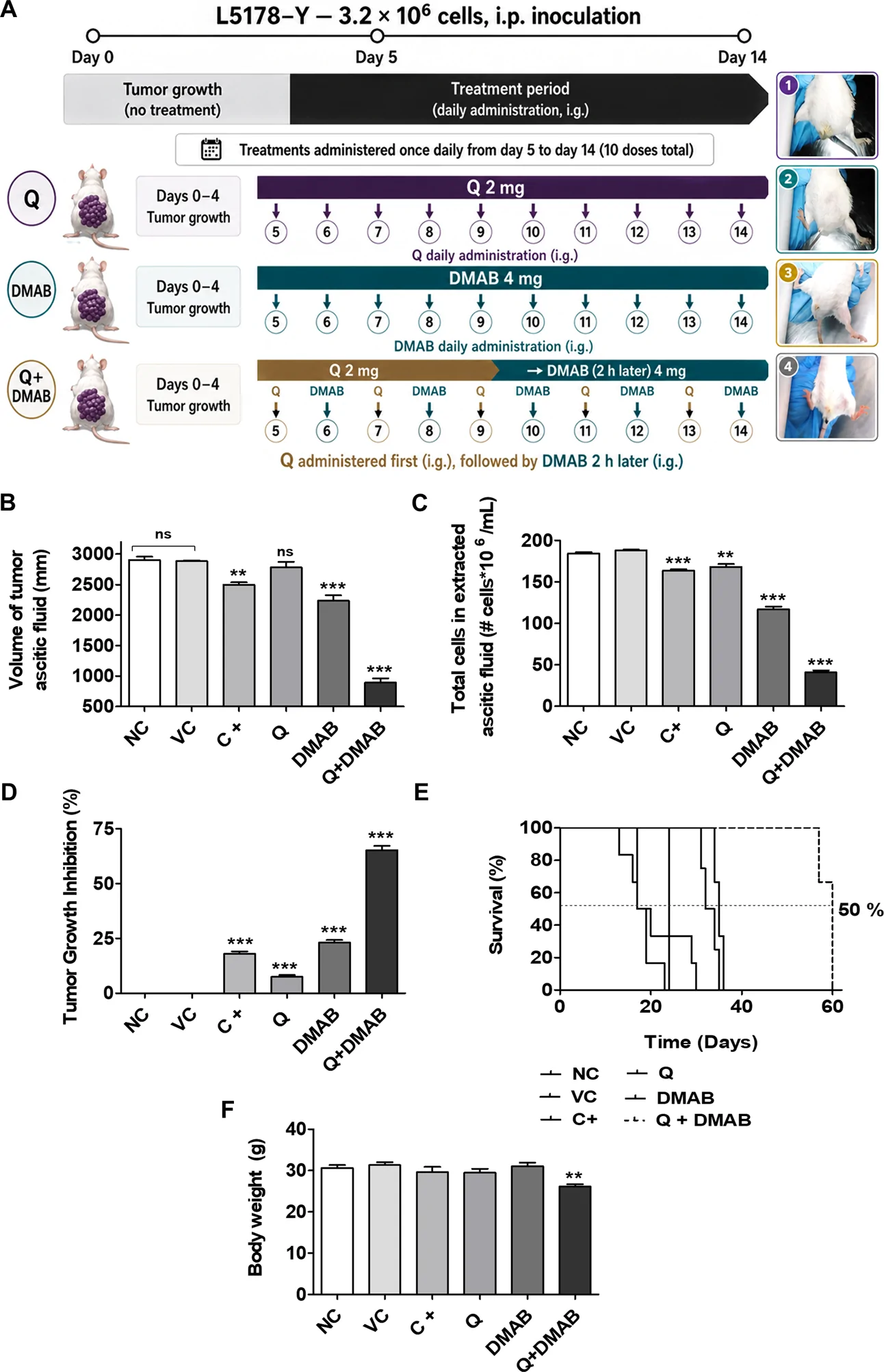
*In vivo* antitumor effects of Q and DMAB in the L5178-Y lymphoma model. (A) Experimental design. BALB/c mice were inoculated intraperitoneally with L5178-Y cells and treated daily by intragastric gavage for 10 days, beginning on day 4 after tumor-cell inoculation. Experimental groups included negative control (NC), vehicle control (VC), Q, DMAB, Q + DMAB, and cyclophosphamide as the positive control (C+); representative animal photographs in panel A are courtesy of G. Carrasco-Torres. Copyright 2025. (B) Ascitic fluid volume. (C) Number of viable tumor cells in ascitic fluid. (D) Tumor growth inhibition (TGI, %). (E) Kaplan–Meier survival curves. (F) Body weight was monitored as an indicator of tumor burden and systemic effects. Data are presented as mean ± SEM from 10 animals per group. Ascitic fluid volume, viable tumor-cell counts, TGI, and body weight were analyzed using one-way ANOVA followed by Tukey’s multiple-comparison test. Survival curves were compared using the log-rank (Mantel–Cox) test. Statistical significance is indicated as ns, not significant; *p < 0.05; **p < 0.01; ***p < 0.001 versus vehicle.

Consistently, analysis of viable tumor cells in ascitic fluid showed a significant decrease in treated groups, with the combination yielding the lowest cell counts among all conditions (Figure 7C).

Tumor growth inhibition (TGI) analysis further supported these findings. The combination achieved the highest inhibitory effect, exceeding that observed with DMAB, Q, and cyclophosphamide, indicating an enhanced antitumor response (Figure 7D).

Kaplan–Meier survival analysis revealed clear differences among treatment groups (Figure 7E). The combination treatment provided the greatest survival benefit, maintaining approximately 50% survival up to day 60. In contrast, control groups showed rapid mortality, while monotherapy groups displayed intermediate survival profiles.

Body weight was monitored as an indicator of overall health status (Figure 7F). Control of animals exhibited increased body weight, consistent with ascitic accumulation. In contrast, the combination-treated group showed reduced body weight, likely reflecting decreased ascitic fluid burden. No treatment-associated body-weight changes suggestive of overt systemic toxicity were observed.

Collectively, these findings indicate that the combination of Q and DMAB enhances antitumor efficacy *in vivo*, as evidenced by reduced tumor burden, decreased viable tumor cell counts, increased tumor growth inhibition, and prolonged survival.

## Discussion

Redox homeostasis has emerged as an important factor influencing tumor progression, therapeutic resistance, and treatment outcomes. Elevated intracellular glutathione (GSH) levels and enhanced antioxidant capacity have been linked to reduced sensitivity to chemotherapy in both murine models and human cancers, including cervical cancer.^8,37–39^ Beyond its role in detoxification, glutathione also plays a key role in regulating tumor cell proliferation, survival, and adaptation to oxidative stress.^38,40,41^ Accordingly, targeting glutathione metabolism and redox balance has gained increasing attention as a potential anticancer strategy.

Mechanistically, the enhanced efficacy of the combined treatment is consistent with a sequential redox sensitization process in which Q pre-exposure may facilitate the subsequent response to DMAB. Q may act as a priming agent, inducing a controlled modulation of intracellular redox balance and partially perturbing glutathione-dependent buffering capacity, thereby lowering the cellular threshold for stress adaptation and increasing susceptibility to subsequent insults. Following this, DMAB exposure further disrupts thiol-dependent systems, likely through interaction with sulfhydryl (−SH) groups, contributing to the observed depletion of intracellular GSH and impairment of thiol-dependent antioxidant defenses. Although the present data directly demonstrate disruption of glutathione homeostasis, they do not establish GSH as the exclusive intracellular target of DMAB. Given the broad thiol reactivity reported for maleamide- and maleimide-related compounds, DMAB may also interact with protein-bound cysteine residues and other low-molecular-weight thiols. Therefore, the observed antitumor effects are more appropriately interpreted as resulting from disruption of a broader thiol-dependent redox network, with glutathione representing a major experimentally demonstrated component. Identification of preferential DMAB-modified proteins will require dedicated thiol-profiling or chemoproteomic analyses. This Q → DMAB sequence establishes a state of redox vulnerability that is not achieved by either monotherapy, underscoring the importance of pharmacological timing in maximizing antitumor efficacy. In agreement with this mechanism, the combined treatment significantly enhanced cytotoxicity, impaired cell cycle progression, promoted apoptosis, and suppressed migratory capacity, while exerting minimal effects on non-tumoral epithelial cells (HaCaT and THLE-3), supporting a selective antitumor response mediated by targeted disruption of glutathione homeostasis.^13,40,42,43^

At the cellular level, the combined treatment produced a pronounced apoptotic response in HeLa cells, as evidenced by the marked increase in TUNEL-positive nuclei and enhanced caspase-3 cleavage. Similar pro-apoptotic effects of Q have been reported in HeLa and other tumor cell models.^44–46^ In parallel, the marked inhibition of wound closure observed in the Q + DMAB group indicates a substantial impairment of migratory capacity. Given the central role of cytoskeletal dynamics and redox signaling in cell adhesion and motility, these findings suggest that disruption of glutathione-dependent homeostasis may affect both tumor cell survival and migration. Although several classes of migration-inhibiting agents are currently under investigation, most remain in preclinical development.^21,47^

Apoptosis induction was further validated by the activation of caspase-3, a key executioner protease responsible for the morphological and biochemical changes associated with programmed cell death.^48^ While all treatments induced some degree of caspase-3 cleavage, the combined Q + DMAB treatment resulted in significantly higher levels of caspase-3 activation, consistent with an enhanced apoptotic response associated with disruption of glutathione-dependent redox homeostasis. These findings align with previous studies showing that GSH depletion sensitizes cancer cells to caspase-dependent apoptosis by lowering the cellular threshold for oxidative and mitochondrial stress.^8,14^

In parallel, combined treatment exerted a pronounced effect on cell cycle dynamics. While Q and DMAB monotherapies induced partial G_0_/G_1_ arrest, their combination promoted a marked accumulation of cells in S phase, accompanied by near-complete depletion of the G_2_/M population. This shift is consistent with altered S-phase progression associated with disruption of redox homeostasis. Notably, nuclear GSH has been implicated in the control of the G_1_-to-S transition and DNA replication fidelity^14,49^ and its depletion may compromise replication processes, leading to S-phase arrest. The divergence from previously reported Q-induced G_2_/M arrest^50,51^ highlights the context-dependent nature of redox-mediated cell cycle regulation and underscores the modulatory effect of combination strategies. Importantly, the pronounced S-phase accumulation induced by Q + DMAB in HeLa cells was not observed in nonmalignant HaCaT cells, which maintained a cell-cycle distribution comparable to that of vehicle-treated controls. This differential response supports the selectivity of the combined treatment and suggests a greater susceptibility of HeLa cells to treatment-associated cell-cycle disruption. Nevertheless, validation in additional nonmalignant and tumor-derived cellular models will be required to determine the broader relevance of this selective response.

An important finding of this study is the dissociation between ROS accumulation and cytotoxicity. While Q reduced steady-state ROS levels and the combined treatment did not induce sustained ROS accumulation, a pronounced depletion of GSH and a reduction in the GSH/GSSG ratio were observed. These findings indicate that cytotoxicity is primarily driven by impairment of redox buffering capacity rather than ROS overload. Importantly, the additional analysis in non-tumorigenic HaCaT cells showed a comparatively attenuated disruption of glutathione-dependent redox balance, with a less pronounced effect on total glutathione content than that observed in HeLa cells. This differential response supports a greater vulnerability of HeLa cells to Q + DMAB-induced disruption of glutathione homeostasis compared with HaCaT cells. This observation aligns with emerging evidence suggesting that depletion of antioxidant reserves is sufficient to trigger cell death in redox-dependent tumors, even in the absence of measurable ROS accumulation.^9,52,53^

Functionally, this redox disruption translated into inhibition of cell migration, indicating an impairment of migratory capacity in HeLa cells. Given the role of cytoskeletal dynamics and redox signaling in cell motility, the observed impairment of migration suggests that glutathione-dependent processes contribute not only to survival but also to the regulation of tumor cell motility. This dual impact reinforces the therapeutic relevance of targeting redox homeostasis.

Importantly, the translational relevance of these findings was supported by *in vivo* evaluation. Combined Q + DMAB treatment significantly reduced ascitic tumor growth, decreased viable tumor cell counts, and achieved superior tumor growth inhibition compared with monotherapies and cyclophosphamide. Moreover, survival analysis demonstrated a marked extension of lifespan, with 50% survival maintained up to approximately day 60. The observed reduction in body weight in the combination group was consistent with decreased ascitic fluid accumulation and was not accompanied by treatment-associated body weight changes suggestive of overt systemic toxicity. These findings indicate that redox-targeted sensitization strategies can produce a meaningful antitumor response *in vivo*.

Notably, the lack of significant antitumor activity observed with Q monotherapy *in vivo* further emphasizes that its primary role in this system is not as a direct cytotoxic agent, but rather as a pharmacological sensitizer that enhances the efficacy of thiol-reactive compounds such as DMAB. This highlights the importance of temporal and mechanistic coordination in combination therapies targeting redox vulnerabilities. These findings position redox-directed sensitization as a rational framework for the design of next-generation combination therapies targeting metabolic vulnerabilities in cancer.

An important limitation of this study is the use of different tumor models for the *in vitro* and *in vivo* evaluations. Mechanistic analyses were performed mainly in HeLa cervical cancer cells, whereas antitumor efficacy was assessed in the murine L5178-Y lymphoma model. Therefore, the *in vivo* results should be interpreted as proof-of-concept evidence rather than direct validation in cervical cancer. Further studies in additional cervical cancer models and other tumor systems are needed to determine the broader applicability of this strategy.

In summary, this study provides mechanistic and functional evidence that sequential modulation of glutathione-dependent redox homeostasis represents a promising strategy to enhance antitumor efficacy. By preferentially disrupting glutathione-dependent redox homeostasis in HeLa cells while producing a comparatively lower impact on nonmalignant epithelial models, this approach supports further investigation of redox-based combination strategies with potential for improved selectivity and translational relevance.

## Conclusion

The present study demonstrates that the combination of Q and 3′,5′-dimaleamylbenzoic acid elicits a pronounced antitumor response associated with disruption of glutathione-dependent redox homeostasis, as the observed effects were linked primarily to intracellular glutathione depletion and a reduced capacity to maintain redox balance rather than to sustained ROS generation. Pre-exposure to Q appears to modulate the intracellular redox environment and increase tumor cell susceptibility to the subsequent thiol-reactive effects of DMAB, while the sequential Q→DMAB strategy enhanced cytotoxicity, promoted S-phase accumulation and apoptosis, and reduced cell migration, with a comparatively lower impact on nonmalignant epithelial cells.

Importantly, these effects were also reflected *in vivo*, where the combined treatment reduced tumor-associated ascitic development, decreased viable tumor cell burden, and extended survival without treatment-associated body weight changes suggestive of overt systemic toxicity. Together, these findings support the concept that targeting glutathione-dependent vulnerabilities through sequential redox modulation may enhance antitumor efficacy and warrants further evaluation in additional tumor models.

## Data Availability

The datasets generated and/or analyzed during the current study are available from the corresponding author upon request.
